# Dental Issues And Depressive Symptoms In Institutionalized Older Adults

**DOI:** 10.1093/geroni/igab046.3129

**Published:** 2021-12-17

**Authors:** Alexandre Bulgarelli, Luiza Guilhermina Lopes, Karla Frichembruder, Camila Santos

**Affiliations:** 1 Federal University of Rio Grande do Sul, Porto Alegre, Rio Grande do Sul, Brazil; 2 University of Rio Grande do Sul, Porto Alegre, Rio Grande do Sul, Brazil; 3 Federal University of Rio Grande do Sil, Porto Alegre, Rio Grande do Sul, Brazil

## Abstract

Objective: To map and discuss scientific knowledge involving the research object "Oral health conditions and depression in institutionalized elderly people". Method: Systematic scoping review. The mapping of the selected data was carried out using Summative Content Analysis from Manifest Themes' perspective in the texts. After the exclusions, 27 articles were selected. Results: With the analysis of the articles, it was possible to divide them into two themes named: ‘Oral health condition: Long-term institution, depressive disorders and pluralities’ and ‘Depression in institutionalized older adults: medicalization, oral health conditions and subjectivities’. Heterogeneity was found in the sub-themes of the articles accessed and in the characteristics of the published studies as well. All continents have publications on the topic. Many studies with deductive methods have been carried out when dealing with its methodology, and less research has been carried out with inductive methods. Conclusion: The present study identified that there is an association between some oral health conditions (dry mouth and tooth loss) and prevalence of depressive disorders in institutionalized polder people. The knowledge produced about oral health and mental health in long-term care facilities for the elderly is relatively recent, reflecting the contemporary nature of the theme. Besides, the construction of this knowledge is associated with the diversity of epidemiological and qualitative studies seeking to answer questions that involve technical and subjective plurality involving mental health and oral health of institutionalized older adults.

